# Supplementary material for: Use of research electronic data capture (REDCap) in a sequential multiple assignment randomized trial (SMART): a practical example of automating double randomization
Source: BMC Med Res Methodol. 2023 Jul 6;23:162. doi: 10.1186/s12874-023-01986-6 (PMC10327314; doi:10.1186/s12874-023-01986-6)
Supplement: Supplementary file 1 — Supplementary Material 1 [file 12874_2023_1986_MOESM1_ESM.docx]

Appendix 1: 1^st^ Randomization Code

Also Accessible at: <https://github.com/cdonelson/double-randomization>

import csv

import requests

import boto3

import os

import json

def pull_consent_records():

data = {

'token': os.environ.get("REDCAP_TOKEN"),

'content': 'record',

'format': 'json',

'type': 'flat',

'csvDelimiter': '',

'rawOrLabel': 'raw',

'rawOrLabelHeaders': 'raw',

'exportCheckboxLabel': 'false',

'exportSurveyFields': 'false',

'exportDataAccessGroups': 'false',

'returnFormat': 'json',

'filterLogic': '[baseline_complete] = "2" AND [consent_2] = "1" AND [randomization1] = ""'

}

r = requests.post(os.environ.get("REDCAP_ENDPOINT",data=data)

print('HTTP Status ' + str(r.status_code) + ": Consenting records downloaded")

return r.json()

def get_csv_s3_lambda(alloc_csv):

ACCESS_KEY = os.environ.get("WINDSOR_ACCESS_KEY")

SECRET_KEY = os.environ.get("WINDSOR_SECRET_KEY")

BUCKET = os.environ.get("WINDSOR_BUCKET")

s3_csv = "allocation-tables/current/" + alloc_csv

alloc_csv = 'tmp/' + alloc_csv

s3 = boto3.client('s3',aws_access_key_id=ACCESS_KEY, aws_secret_access_key=SECRET_KEY)

s3.download_file(BUCKET, s3_csv, alloc_csv)

return

def load_allocation_table(csv_to_load):

data_table = []

get_csv_s3_lambda(csv_to_load)

with open(csv_to_load, 'r') as csvfile:

csvreader = csv.reader(csvfile)

fields = next(csvreader)

for row in csvreader:

data_table.append(row)

return (data_table, fields)

def save_allocation_table_lambda(local_filename, alloc_table, alloc_fields):

with open("tmp/" + local_filename, 'w', newline='') as csvfile:

spamwriter = csv.writer(csvfile, delimiter=',')

spamwriter.writerow(alloc_fields)

for row in alloc_table:

spamwriter.writerow(row)

return

def push_to_s3(alloc_csv_new):

ACCESS_KEY = os.environ.get("WINDSOR_ACCESS_KEY")

SECRET_KEY = os.environ.get("WINDSOR_SECRET_KEY")

BUCKET = os.environ.get("WINDSOR_BUCKET")

s3_csv = "allocation-tables/current/" + alloc_csv_new

s3 = boto3.client('s3',aws_access_key_id=ACCESS_KEY, aws_secret_access_key=SECRET_KEY)

try:

s3.upload_file("tmp/" + alloc_csv_new, BUCKET, s3_csv)

print("New allocation table upload successful")

except:

print("New allocation table failed to upload")

def first_randomization(consent_list, alloc_table, alloc_fields):

tagged_consent = []

for record in consent_list:

if alloc_table[0][1] == "Navigation":

label = '1'

elif alloc_table[0][1] == "Referral":

label = '2'

else:

print("Unrecognized treatment for record_id: " + record['record_id'])

label = ''

record['randomization1'] = label

record['st_intervention_complete'] = '2'

record['covid_test_complete'] = ""

record['nd_intervention_complete'] = ""

tagged_consent.append(record)

alloc_table.append(alloc_table.pop(0))

save_allocation_table_lambda("randomization_stage_1.csv", alloc_table, alloc_fields)

push_to_s3("randomization_stage_1.csv")

return tagged_consent

def push_to_redcap(consent_list):

import_json = json.dumps(consent_list)

data = {

'token': os.environ.get("REDCAP_TOKEN"),

'content': 'record',

'format': 'json',

'type': 'flat',

'overwriteBehavior': 'normal',

'forceAutoNumber': 'false',

'data': import_json,

'returnContent': 'count',

'returnFormat': 'json'

}

r = requests.post(os.environ.get("REDCAP_ENDPOINT"),data=data)

print('HTTP Status ' + str(r.status_code) + ": Records imported to REDCap")

def lambda_handler(context, event):

# Pull in all records who have consented 'Yes' from REDCap

consent_list = pull_consent_records()

# Load in first allocation table from S3

alloc_table, alloc_fields = load_allocation_table("randomization_stage_1.csv")

# Perform first randomization on each record in consent_list and store new allocation table in S3

tagged_consent = first_randomization(consent_list, alloc_table, alloc_fields)

# Push first randomization results to REDCap

push_to_redcap(consent_list)

Appendix 2: 2^nd^ Randomization Code

Also Accessible at: <https://github.com/cdonelson/double-randomization>

import csv

import requests

import boto3

import os

import json

def pull_for_second_rand():

data = {

'token': os.environ.get("REDCAP_TOKEN"),

'content': 'record',

'format': 'json',

'type': 'flat',

'csvDelimiter': '',

'rawOrLabel': 'raw',

'rawOrLabelHeaders': 'raw',

'exportCheckboxLabel': 'false',

'exportSurveyFields': 'false',

'exportDataAccessGroups': 'false',

'returnFormat': 'json',

'filterLogic': '[randomization1] != "" AND [randomization2] = "" AND [st_intervention_complete] = "2" AND [covidtest] != "" AND [covid_test_complete] = "2"'

}

r = requests.post(os.environ.get("REDCAP_ENDPOINT"),data=data)

print('HTTP Status ' + str(r.status_code) + ": Records download successful")

return r.json()

def get_csv_s3_lambda(alloc_csv):

ACCESS_KEY = os.environ.get("WINDSOR_ACCESS_KEY")

SECRET_KEY = os.environ.get("WINDSOR_SECRET_KEY")

BUCKET = os.environ.get("WINDSOR_BUCKET")

s3_csv = "allocation-tables/current/" + alloc_csv

alloc_csv = 'tmp/' + alloc_csv

s3 = boto3.client('s3',aws_access_key_id=ACCESS_KEY, aws_secret_access_key=SECRET_KEY)

s3.download_file(BUCKET, s3_csv, alloc_csv)

return

def load_allocation_table(csv_to_load):

data_table = []

get_csv_s3_lambda(csv_to_load)

with open(csv_to_load, 'r') as csvfile:

csvreader = csv.reader(csvfile)

fields = next(csvreader)

for row in csvreader:

data_table.append(row)

return (data_table, fields)

def save_allocation_table_lambda(local_filename, alloc_table, alloc_fields):

with open("tmp/" + local_filename, 'w', newline='') as csvfile:

spamwriter = csv.writer(csvfile, delimiter=',')

spamwriter.writerow(alloc_fields)

for row in alloc_table:

spamwriter.writerow(row)

return

def push_to_s3(alloc_csv_new):

ACCESS_KEY = os.environ.get("WINDSOR_ACCESS_KEY")

SECRET_KEY = os.environ.get("WINDSOR_SECRET_KEY")

BUCKET = os.environ.get("WINDSOR_BUCKET")

s3_csv = "allocation-tables/current/" + alloc_csv_new

s3 = boto3.client('s3',aws_access_key_id=ACCESS_KEY, aws_secret_access_key=SECRET_KEY)

try:

s3.upload_file("tmp/" + alloc_csv_new, BUCKET, s3_csv)

print("New allocation table upload successful")

except:

print("New allocation table failed to upload")

def second_randomization(second_consent, alloc_table, alloc_fields):

tagged_second_consent = []

for record in second_consent:

for row in alloc_table:

if record['randomization1'] == "1" and record['covidtest'] == "1" and record['covidtestresult'] == "1" and row[0] == "Navigation/Tested Positive":

record['randomization2'] = row[2]

alloc_table.append(alloc_table.pop(alloc_table.index(row)))

break

elif record['randomization1'] == "1" and record['covidtest'] == "1" and record['covidtestresult'] == "2" and row[0] == "Navigation/Tested Negative":

record['randomization2'] = row[2]

alloc_table.append(alloc_table.pop(alloc_table.index(row)))

break

elif record['randomization1'] == "1" and record['covidtest'] == "0" and row[0] == "Navigation/Not Tested":

record['randomization2'] = row[2]

alloc_table.append(alloc_table.pop(alloc_table.index(row)))

break

elif record['randomization1'] == "2" and record['covidtest'] == "1" and record['covidtestresult'] == "1" and row[0] == "Referral/Tested Positive":

record['randomization2'] = row[2]

alloc_table.append(alloc_table.pop(alloc_table.index(row)))

break

elif record['randomization1'] == "2" and record['covidtest'] == "1" and record['covidtestresult'] == "2" and row[0] == "Referral/Tested Negative":

record['randomization2'] = row[2]

alloc_table.append(alloc_table.pop(alloc_table.index(row)))

break

elif record['randomization1'] == "2" and record['covidtest'] == "0" and row[0] == "Referral/Not Tested":

record['randomization2'] = row[2]

alloc_table.append(alloc_table.pop(alloc_table.index(row)))

break

record['nd_intervention_complete'] = "2"

# Convert randomized treatement into REDCap values

if record['randomization2'] == "Navigation":

record['randomization2'] = "1"

elif record['randomization2'] == "Brief Counseling":

record['randomization2'] = "2"

elif record['randomization2'] == "Critical Dialogue":

record['randomization2'] = "3"

elif record['randomization2'] == "Brochure":

record['randomization2'] = "4"

else:

record['randomization2'] = ""

tagged_second_consent.append(record)

save_allocation_table_lambda("randomization_stage_2.csv", alloc_table, alloc_fields)

push_to_s3("randomization_stage_2.csv")

return tagged_second_consent

# Pull in records with a non-null first randomization and covidtest

second_consent = pull_for_second_rand()

# Load in second allocation table from S3

alloc_table, alloc_fields = load_allocation_table("randomization_stage_2.csv")

# Perform second randomization on each record in second_second and store new allocation table in S3

tagged_second_consent = second_randomization(second_consent, alloc_table, alloc_fields)

# Push second randomization results to REDCap

push_to_redcap(tagged_second_consent)
